# Comparative mediation analysis of dog and cat ownership and perceived stress

**DOI:** 10.1371/journal.pone.0350098

**Published:** 2026-06-17

**Authors:** Niwako Ogata, Anna L. Heck, Hsin-Yi Weng

**Affiliations:** 1 Department of Veterinary Clinical Sciences, College of Veterinary Medicine, Purdue University, West Lafayette, United States of America; 2 Department of Animal Sciences, College of Agriculture, Purdue University, West Lafayette, United States of America; 3 Department of Comparative Pathobiology, College of Veterinary Medicine, Purdue University, West Lafayette, United States of America; Mansheyet El Bakry General Hospital, EGYPT

## Abstract

Companion animals, such as dogs and cats, are widely reported to be associated with lower stress, yet species-specific differences remain unclear. We conducted a comparative mediation analysis to examine how pet ownership type (dog vs. cat) relates to perceived stress, leveraging a large, balanced dataset (1,923 dog owners and 1,804 cat owners) collected throughout the COVID-19 pandemic. Investigated mediators included human-animal interaction, emotional closeness, perceived costs, behavior problems, and disease transmission concerns. We used a directed acyclic graph (DAG)-guided, bootstrap-based path modeling approach to estimate mediation-based associations. Dog and cat ownership differed only marginally in their overall association with perceived stress, with dog ownership associated with slightly lower stress levels. Comparative mediation analyses revealed the largest species-specific difference in human-animal interaction. This mediator showed a strong inverse association with perceived stress for dog ownership but only a minimal association for cat ownership. Accordingly, human-animal interaction emerged as the most influential mediator for dog ownership, whereas emotional closeness was most influential for cat ownership. Moreover, emotional closeness showed a positive association with perceived stress in both groups. Perceived costs and behavior problems showed the strongest associations with higher perceived stress across species, slightly more pronounced for dog ownership. Yet their mediation-based associations were weak, largely attributable to minimal differences in perceived costs and behavior problems between dog and cat ownership. Only 26% of the overall association between ownership type and perceived stress was accounted for by the study mediators, indicating the need to explore additional mediators. This study advances understanding of how the human-animal bond is associated with perceived stress and underscores species-specific differences in these associations. These findings can help generate hypotheses for future longitudinal studies and may help inform the development of species-tailored interventions.

## Introduction

Companion animals are widely believed to be associated with lower stress and better psychological wellbeing. [1,2] Theoretical frameworks such as social support theory and attachment theory propose mechanisms through which companion animals may be associated with stress, including emotional support, tactile comfort, opportunities for nurturance, and feelings of security. [3–7] Numerous experimental studies have demonstrated that companion animals can influence physiological markers of stress, such as cortisol, oxytocin, and cardiovascular measures. [8–12] Additional experimental studies have shown that direct interaction with one’s own dog following a stressor accelerated physiological and psychological recovery. [13–15] Collectively, these findings suggest that companion animals may buffer against stress, potentially through both biological and psychological pathways. [16]

While experimental studies provide compelling evidence for the potential benefits of companion animals, findings from observational research in real-world contexts are more nuanced. Evidence for stress-buffering effects of companion animals remains inconsistent in stressful situations such as the COVID-19 pandemic and natural disasters. [17,18] The human-animal bond in these settings is complex and multifaceted; although it may buffer stress, it can also introduce additional challenges and uncertainties, such as financial burden, limited access to veterinary care, and concerns about disease transmission. [19,20] Moreover, these events may be experienced differently by dog and cat owners. For instance, the lockdown period of the COVID-19 pandemic might have impacted dogs and their owners more substantially than cats and their owners, as many outdoor activities and social events were prohibited. [21] At the same time, some dogs, especially those prone to separation anxiety, may have benefited from increased owner presence at home. [22] Despite these challenges, studies found that most owners valued the established human-animal bond and demonstrated resilience during crises. [18–20,23,24] However, disruptions to daily routines and restricted access to veterinary care and other support services further highlight the need for interventions and support systems that not only foster resilience but also address the practical challenges of maintaining positive human-animal bonds during times of crisis. [25,26]

The human–animal relationship is inherently complex and closely tied to sociodemographic factors such as gender, age, marital status, financial status, and personality. [27,28] Failure to account for these factors can lead to biased conclusions in observational studies, where random assignment to pet ownership is neither feasible nor ethical. Causal knowledge is, therefore, a prerequisite to make inferences from observational studies. [29,30] Here, we employed a directed acyclic graph (DAG) framework to transparently present our study’s causal assumptions. The DAG was then systematically analyzed to identify confounders for adjustment, clarify assumed causal pathways, and guide statistical analysis. [31,32] This approach represents a methodological advance, as few studies in this domain have used a DAG-guided comparative mediation analysis to examine complex relationships between pet ownership and psychological outcomes.

Given the multifaceted nature of pet effects on stress, it becomes especially important to investigate the constructs underlying these association patterns. Despite broad interest in the topic, few studies directly compare dog and cat ownership, even though species-specific differences in physiological and psychological outcomes have been reported. [33–35] Existing research generally finds stronger emotional closeness, greater social facilitation, and more consistent physical and mental health benefits associated with dogs compared with cats. [27,36] Dog owners also tend to experience higher physical activity levels and perceive their pets as more integrated into family life. [4,35] In contrast, cat–owner relationships, while meaningful, are less frequently characterized by the same degree of emotional intensity or social engagement. [37] Factors such as emotional closeness, human–animal interaction, perceived cost of care, and concerns about disease transmission may mediate the relationship between pet ownership and stress. [3,27,38] Understanding these mediation-informed association patterns is essential for clarifying species-specific differences, yet comparative mediation research in this area is virtually absent.

To address these gaps, this study aims to characterize how dog versus cat ownership among U.S. adults during the COVID-19 pandemic relates to perceived stress. We examined mediation-informed associations involving positive factors (human-animal interaction and emotional closeness) and negative factors (perceived cost of care, disease transmission concerns, and pet behavior problems). We hypothesized that pet ownership type is associated with perceived stress and that dog ownership would be associated with lower perceived stress than cat ownership. We further hypothesized that increased human-animal interaction and emotional closeness would be associated with lower stress, while higher perceived costs, concerns about disease transmission, and behavior problems would be associated with higher stress. By integrating a DAG-guided mediation analysis with a comparative evaluation of dog and cat ownership, this study addresses critical gaps in the literature and provides a foundation for future research.

## Materials and methods

### Data source and study population

Data for this study were extracted from a longitudinal project conducted by our team between June 2020 and September 2024. The original study aimed to examine the impact of the COVID-19 pandemic on human-animal relationships, pet behaviors, and mental health. Participants were recruited using CloudResearch® (formerly TurkPrime; Prime Research Solutions LLC), an online crowdsourcing platform for behavioral and longitudinal research. [39] This approach enabled efficient, remote recruitment and surveying of eligible participants across the United States. It also allowed for follow-up surveys while maintaining anonymity and avoiding the collection of personally identifying information (e.g., name and other contact information). Participation required informed electronic consent. Participants were shown an online consent form and had to click ‘Yes’ to proceed; those who did not consent could not access the survey. The original longitudinal study from which these data were extracted was approved under an exemption [Category 2(iii)] by the Purdue University Institutional Review Board (IRB-2020–760 and IRB-2024–1012).

Eligible participants were U.S. residents aged 18 years or older who owned one or more dogs and/or cats (but no other pet types) at recruitment. For those with multiple dogs or cats, pet-related questions were answered for the pet to which the participant felt most attached. Participants completed an initial survey and were categorized into dog or cat groups based on the pet selected.

Six cohorts were followed between June 2020 and July 2022. Two additional cohorts, recruited in June 2023 and September 2024, were not followed longitudinally. For analysis, the study period was divided into four pandemic phases: Lockdown, Reopening, Recovery, and Stabilization, corresponding to major public health and societal changes. Additional details on the original study design and procedures are provided in Ogata et al. (2023) and Weng et al. (2024). [18,22]

In this study, only initial survey responses were used, yielding eight cross-sectional datasets. To avoid contamination across ownership types, we included only single-species pet owners in the study. Table 1 summarizes the number of respondents by cohort, pet ownership type and pandemic phase.

**Table 1:** Number of study participants by cohort, pet ownership type, survey date, and pandemic phase.

	Number of participants			
Cohort	Dog	Cat	Survey date	Phase
1	446	469	June/2020	Lockdown
2	363	291	January/2021	Reopening
3	57	58	April/2021	Recovery
4	196	178	September/2021	Recovery
5	155	143	December/2021	Recovery
6	126	126	July/2022	Recovery
7	251	193	June/2023	Stabilization
8	329	346	September/2024	Stabilization
Total	1923	1804		

### Study variables

#### Outcome.

The primary outcome for this study was perceived stress, measured using the 10-item Perceived Stress Scale (PSS). [40] Respondents rated how often they experienced certain feelings and thoughts during the past month on a scale from 0 (never) to 4 (very often). Total PSS scores ranged from 0 to 40, with higher scores indicating greater perceived stress.

#### Mediators.

Investigated mediators of the relationship between pet ownership type and perceived stress included owner-pet relationships, behavior problems in pets, and disease transmission concerns. Based on prior research, these variables were hypothesized to lie downstream of pet ownership and to be linked to perceived stress. [35,41–46]

Owner-pet relationships were assessed using the Dog Owner Relationship Scale (DORS) and the Cat-Owner Relationship Scale (CORS), [47] both adapted from the Monash Dog-Owner Relationship Scale (MDORS). [48] In the CORS, items reflecting dog-specific behaviors (e.g., taking pets in the car and visiting people) were removed, and items specific to cat-owner relationship (e.g., appreciation of the cat’s independence) were added. Three subscales were evaluated: human-animal interaction (HAI), emotional closeness (Emotion), and perceived costs (Cost). Several items were rearranged between the HAI and Emotion subscales in the CORS compared with the DORS. Scores for each subscale, calculated as the means of relevant items, ranged from 1 to 5, with higher HAI and Emotion scores indicating closer relationships, and higher Cost scores reflecting greater perceived burden.

Behavior problems were measured by the number of reported categories (general aggression, fear-induced aggression, fear and anxiety, separation anxiety, and house soiling), with participants indicating the presence or absence of each category. Scores ranged from 0 (no reported behavior problems) to 5 (all categories reported).

Concerns about disease transmission were rated on a 5-point scale, with higher scores indicating greater concern about transmission from owner to pet and vice versa.

#### Other covariates.

Additional variables were collected to account for potential confounding. Demographic and socioeconomic data included age, gender, marital status, income, housing type, and household composition. Owner’s personality traits were assessed using the Five-Factor Model (Big Five). [49] Pet characteristics such as sex, age, breed, and ownership duration were obtained. Lastly, COVID-related variables encompassed phase, routine changes, and high risk for contracting COVID-19.

### Causal assumptions

**Fig 1:**
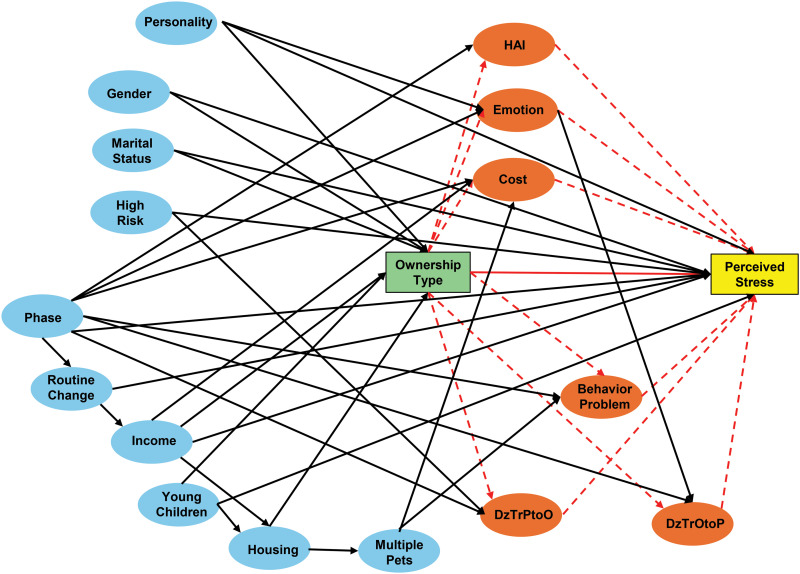
Directed acyclic graph illustrating the hypothesized causal structure used to identify confounders for the association between pet ownership type and perceived stress. Orange nodes represent mediators; blue nodes represent covariates. Solid red lines indicate the direct paths from pet ownership type to perceived stress; dashed red lines indicate indirect paths through mediators; black lines show hypothesized associations among covariates, the exposure, mediators, and the outcome.

To guide confounder identification and statistical adjustment for our mediation analysis, we constructed a directed acyclic graph (DAG) to make explicit our assumed causal structure among pet ownership type, mediators, covariates, and perceived stress (Fig 1). [50] In DAGs, variables are represented as nodes and hypothesized causal relationships as directed arrows.

Drawing on prior studies, we considered human personality traits, gender, marital status, income, presence of children under 3 years old in the household, housing type, and ownership of multiple pets as direct determinants of pet ownership type. [27,28,51,52] We also assumed that pandemic phase, high risk for contracting COVID-19, and routine changes affect perceived stress through both direct and indirect pathways. [18,20]

The DAG was used to identify minimally sufficient adjustment sets for estimating adjusted associations along the specified paths; however, these assumptions do not imply that causal effects can be established with our cross-sectional data.

### Statistical analysis

We performed a DAG-guided path analysis to model the association between pet ownership type (dog vs. cat) and perceived stress. The analysis partitioned the overall association into components corresponding to the specified direct path and mediator-related paths. For each mediator-based association, two paths were modeled: from pet ownership type to mediator (a-path) and from mediator to perceived stress (b-path). For each modeled path, a minimally sufficient set of confounders for statistical adjustment was identified by analyzing the DAG using the R package “dagitty” (Table 2). [31] When multiple adjustment sets were identified, we used the set that maximized the number of complete cases.

**Table 2:** Confounders used for statistical adjustment for each modeled path relating pet ownership type to perceived stress, identified from the study DAG (Fig 1).

	Confounder set
Direct path (ownership type → stress)	gender, personality, income, marital status, multiple pets, young children
Mediator-related paths^a^	
ownership type → human-animal interaction → stress	a-path: pandemic phase b-path: ownership type, pandemic phase
ownership type → emotional closeness → stress	a-path: personality, pandemic phase b-path: ownership type, personality, pandemic phase
ownership type → perceived costs → stress	a-path: income, multiple pets b-path: ownership type, pandemic phase, income, multiple pets
ownership type → behavior problem → stress	a-path: pandemic phase, multiple pets b-path: ownership type, pandemic phase, multiple pets
ownership type → owner-to-pet transmission → stress	a-path: personality, pandemic phase b-path: ownership type, pandemic phase, emotional closeness
ownership type → pet-to-owner transmission → stress	a-path: pandemic phase b-path: ownership type, pandemic phase, high risk for contracting COVID-19

^a^Each mediator-related path is modeled with the a-path (ownership type → mediator) and the b-path (mediator → stress)

Specified paths were estimated using a DAG-guided, bootstrap-based path modeling approach. To evaluate whether mediator-based association differed between dog and cat ownership, we tested interaction terms between pet ownership type and each mediator. If an interaction term was statistically significant, we reported species-specific mediation-based association.

The study outcome and mediators were re-scaled using the z-score method for comparability of results across them. Statistical inference was based on 5,000 bootstrap replicates to derive 95% confidence intervals (CIs). Missing data were handled using Multiple Imputation by Chained Equation (MICE), with 20 imputations and 20 iterations per imputation. [53] Analyses were repeated on imputed datasets and pooled using Rubin’s rules. Sensitivity analyses were performed by comparing complete-case and imputed results for all key estimates. All analyses were performed in RStudio (Version 2025.09.0; Posit Team, 2025).

## Results

A total of 1,923 dog owners (52%) and 1,804 cat owners (48%) were included in the study, yielding a large and balanced sample for comparative analysis. Table 3 summarizes the sociodemographic, pet-related, and COVID-related characteristics of participants, stratified by pet ownership type. The sample encompasses a wide range of demographic and socioeconomic backgrounds, pet characteristics, as well as key mediators and pandemic-related factors, which may improve the applicability of findings to similar U.S. pet owners.

**Table 3:** Summary statistics of study participants and study variables by pet ownership type (dog vs. cat).

Variables		Dog	Cat
**Sociodemographic**			
Gender			
	Woman	1,027 (54%)	962 (54%)
	Man	869 (46%)	810 (46%)
Marital status			
	Married/Partnered	871 (58%)	703 (50%)
	Not married/partnered	632 (42%)	693 (50%)
Income			
	Low	528 (36%)	610 (44%)
	Middle	599 (40%)	533 (38%)
	High	358 (24%)	244 (18%)
Housing type			
	Apartment	336 (18%)	540 (30%)
	House with fences	981 (51%)	600 (33%)
	House without fences	546 (28%)	606 (34%)
	Other	53 (2.8%)	58 (3.2%)
Children under 3 years old			
	No	1,531 (80%)	1,420 (79%)
	1	276 (14%)	257 (14%)
	>1	116 (6.0%)	127 (7.0%)
Multiple pets			
	No	1,286 (67%)	1,004 (56%)
	Yes	637 (33%)	800 (44%)
**Mediators**			
Human-animal interaction		3.6 (3.1–4.0)	4.7 (3.8–5.0)
Emotional closeness		4.0 (3.4–4.5)	3.6 (3.1–4.2)
Perceived costs		1.8 (1.2–2.6)	1.7 (1.2–2.6)
Number of behavior problems		1 (0–2)	1 (0–2)
Infection concern (owner to pet)		2 (1–3)	2 (1–4)
Infection concern (pet to owner)		1 (1–3)	1 (1–3)
**COVID related**			
Pandemic phase			
	Lockdown	446 (23%)	469 (26%)
	Reopening	363 (19%)	291 (16%)
	Recovery	534 (28%)	505 (28%)
	Stabilization	580 (30%)	539 (30%)
Routine changes			
	No	922 (50%)	878 (51%)
	Yes	914 (50%)	850 (49%)
High risk for COVID			
	No	1,136 (74%)	1,077 (76%)
	Yes	400 (26%)	330 (23%)

Continuous variables are presented as median (inter-quartile range [IQR]); categorical variables are shown as frequency (%). Note: Sample sizes may vary across variables due to missing values.

We tested our hypotheses by comparing perceived stress between dog and cat ownership, and then decomposing the overall association into direct and mediator-related paths. Mediator-based associations were evaluated for each hypothesized path, with species-specific estimates reported where statistically significant interactions were detected.

Consistent with our hypothesis, dog ownership was associated with lower perceived stress compared to cat ownership (overall association: −0.054, 95% CI: −0.110, 0.001). Mediation analysis revealed that only approximately 26% of the overall association was attributable to the mediator-based association. Interestingly, the direct path was negatively associated with perceived stress, whereas the joint mediator-based association was positive, with cat ownership as the reference.

**Fig 2:**
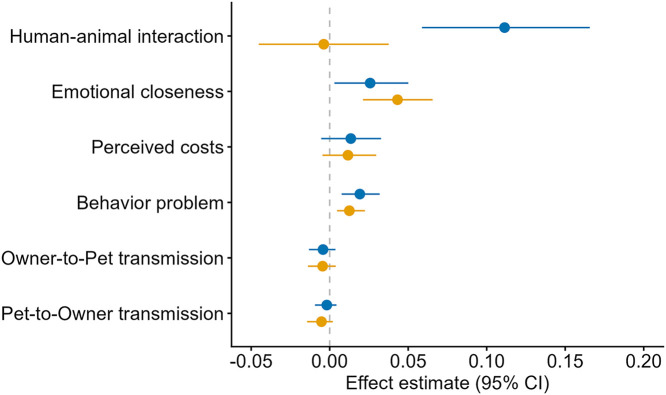
Mediator-based associations with perceived stress, by pet ownership type. Blue: dog ownership; Orange: cat ownership. Error bars indicate 95% confidence intervals. The dashed gray line marks the null value.

Species-specific comparisons showed statistically significant differences in mediator-based associations for HAI, which showed a much stronger association for dog ownership compared to cat ownership (adjusted p = 0.002). For dog ownership, HAI contributed the largest mediator-based association, followed by emotional closeness, perceived costs, and behavior problems; each was associated with higher perceived stress (Fig 2). For cat ownership, emotional closeness was the strongest mediator, followed by perceived costs and behavior problems. Mediator-related paths involving disease transmission concerns were minimal for both pet ownership types.

Partitioning mediator-based associations into the a-path (pet ownership type to mediator) and the b-path (mediator to perceived stress) helps clarify the species-specific mediator-related paths through which pet ownership may be associated with psychological wellbeing (Table 4). The mediator-related path is quantified as the product of the a-path and b-path coefficients, representing the extent to which pet ownership type is associated with perceived stress indirectly through a given mediator. For example, the minimal mediator-based association observed for concern about disease transmission from owner to pet was primarily driven by small a-path coefficients, reflecting negligible differences in disease transmission concern between dog and cat ownership, despite stronger associations with perceived stress reflected in larger b-path coefficients.

**Table 4:** Partition of mediator-based associations into a-path (pet ownership type to mediator) and species-specific b-path (mediator to perceived stress) coefficients (95% CI).

	a-path	b-path (Dog)	b-path (Cat)
HAI	−0.915 (−0.967, −0.863)	−0.122 (−0.178, −0.064)	0.004 (−0.041, 0.049)
Emotion	0.471 (0.406, 0.536)	0.055 (0.006, 0.104)	0.092 (0.046, 0.136)
Cost	0.046 (−0.018, 0.112)	0.292 (0.234, 0.349)	0.252 (0.193, 0.313)
BehPb	0.105 (0.043, 0.168)	0.183 (0.141, 0.223)	0.119 (0.078, 0.164)
O-to-P	−0.037 (−0.108, 0.032)	0.114 (0.064, 0.163)	0.120 (0.070, 0.171)
P-to-O	0.093 (0.039, 0.146)	−0.020 (−0.086, 0.047)	−0.056 (−0.135, 0.023)

a-path: cat ownership is the reference. HAI: human-animal interaction; Emotion: emotional closeness; Cost: perceived costs; BehPb: behavior problem; O-to-P: owner-to-pet transmission concern; P-to-O: pet-to-owner transmission concern.

The direction (sign) of the path coefficients explained why most mediator-related paths were associated with higher perceived stress when cat ownership served as the reference. For instance, the b-path of −0.122 for HAI in dog ownership indicated that higher HAI scores were associated with lower perceived stress. However, dog owners, on average, reported lower HAI scores compared to cat owners (a-path = −0.915), which consequently resulted in the mediator-related path through HAI being associated with increased perceived stress for dog ownership when cat ownership served as the reference. In contrast, the HAI-mediated association for cat ownership was negligible because the b-path was near zero (0.004), reflecting a weak association between HAI and perceived stress among cat owners.

Similarly, dog owners reported higher levels of emotional closeness, perceived costs, and behavior problems than cat owners. As hypothesized, greater perceived costs and more behavior problems were linked to higher perceived stress for both ownership types. Unexpectedly, higher Emotion scores were also linked to increased stress in both groups.

Sensitivity analyses comparing complete-case and imputed datasets yielded consistent results, supporting the robustness of the findings. For clarity, only complete-case results are presented.

## Discussion

Recent research highlights that simplistic comparisons of pet owners and non-owners overlook the complexity of the human-animal relationship and its association with psychological wellbeing. [35] Addressing this gap, we applied a DAG-guided comparative mediation analysis to examine how pet ownership type (dog vs. cat) relates to perceived stress, leveraging a large, balanced dataset consisting of cohorts spanning multiple phases of the COVID-19 pandemic. By partitioning the mediator-based associations into the a-path (pet ownership type to mediator) and b-path (mediator to perceived stress), we focused on associations involving owner-pet relationship dimensions (human-animal interaction, emotional closeness, and perceived costs), pet behavior problems, and disease transmission concerns. These mediators were selected based on prior evidence suggesting their central role in the human-animal bond and their potential association with psychological outcomes during public health crises. [35,41–46]

Dog and cat ownership showed only modest differences in their overall association with perceived stress, with dog ownership linked to slightly lower stress levels. The investigated mediators collectively accounted for roughly one-quarter of the overall association, and the combined mediator-related paths were in the opposite direction to the direct path.

Comparative mediation analysis revealed that the largest species-specific difference was in human-animal interaction (HAI). HAI emerged as the strongest mediator for dog ownership, whereas emotional closeness (Emotion) was most influential for cat ownership. Higher HAI scores were associated with lower perceived stress only among dog owners, while higher Emotion scores were linked to higher stress for both species. These patterns suggest species-specific associations between the human-animal bond and psychological outcomes.

Examination of the a- and b-path coefficients further clarifies these species differences. Dog owners reported lower HAI and higher Emotion scores than cat owners, which may partly reflect inherent differences in the Dog Owner Relationship Scale (DORS) and Cat-Owner Relationship Scale (CORS). Although both scales share a core set of items, species-specific items are included in the HAI and Emotion subscales to reflect the unique ways people interact with and perceive their bonds with different species. For example, the questions asking about the frequency of taking the pet on car rides and frequency of taking the pet to visit other people are included in the HAI subscale for dog ownership only, whereas the questions asking about the frequency of the pet sitting on the owner’s lap and frequency of giving treats to the pet are included as the primary HAI items for cat ownership. The Emotional subscale captures affectional bonding and psychological attachment, with minor adaptations for species relevance. Despite that both subscales were standardized using z-scores, item content and pandemic-related restrictions (e.g., limited opportunities for dog-related HAI activities during lockdown) could have influenced observed differences between dog and cat ownership.

These findings align with social support and attachment theories, which posit that human-animal relationships may be associated with psychological wellbeing through both interpersonal and affective mechanisms. The much stronger association between HAI and perceived stress for dog ownership suggests that dog-related activities, such as taking the dog to visit people or going for rides, may coincide with broader social engagement and community integration. [54,55] These forms of interaction often create opportunities for human-human contact, which may be associated with greater perceived social support and lower stress. In contrast, typical HAI for cat ownership, such as lap sitting or treat giving, occurs primarily in the home and may not offer the same degree of opportunity for human-human interactions as dog ownership. The mediation analysis also suggested that the association between cat ownership and perceived stress may be more closely linked to emotional regulation, comfort, and the provision of a stable affective presence, aligning with affective mechanisms proposed by attachment theory. [56]

Notably, the difference in the association between emotional closeness and perceived stress is negligible between dog and cat ownership, and unlike HAI, emotional closeness demonstrated a similar positive association with perceived stress across species, suggesting that emotional closeness may not uniformly buffer stress. The positive association between Emotion and perceived stress is consistent with mixed findings in the broader literature. A systematic review found that higher attachment was associated with better mental health in 15 studies, worse mental health in 22, and yielded mixed or null results in 69 others. [57] Zilcha-Mano et al. (2011) applied the attachment theory framework to show individual variations in attachment anxiety and avoidance, which consequently influenced owner-pet relationships and mental health. [58] Our finding that higher Emotion scores were associated with higher levels of perceived stress may reflect attachment insecurity and anxiety, such as grief, unmet expectations, or intensified worrying. [35,59] Additionally, prior research suggests a nonlinear relationship between emotional attachment and stress, with potential threshold effects that merit further investigation using more robust measurements of pet attachment. [60]

Most observed associations between mediators and perceived stress were consistent with prior research. Perceived costs and behavior problems showed the strongest associations with higher perceived stress in both ownership groups, slightly more pronounced for dog ownership. Cost and behavior problems are well-recognized barriers to a positive owner-pet relationship and are associated with higher stress. [9,27,35,44,61] Owners with strong attachment may be particularly vulnerable to the distress associated with behavioral challenges or financial strain. Additionally, disruptions during COVID-19, such as changes in daily routines, limited access to veterinary care, and reduced social engagement, may have exacerbated behavior problems and caregiving demands, which could be associated with higher perceived stress. [20,22]

Concern about disease transmission from owner to pet was positively associated with perceived stress, whereas concern about disease transmission from pet to owner was not. Our causal structure hypothesized that emotional closeness was associated with concern about disease transmission from owner to pet, and being at high risk for COVID-19 was associated with concern about contracting disease from a pet. After adjusting for these covariates, concern about disease transmission from owner to pet remained associated with perceived stress in both groups, but the association between concern about contracting disease from the pet and stress diminished. This asymmetry suggests that perceived stress is more strongly associated with perceived responsibility for the pet’s wellbeing than with fear of zoonotic infection. This pattern aligns with evidence that many owners view pets as family members. Worries about harming them can evoke guilt and vigilance analogous to caregiver burden in human caregiving contexts. [61,62] During a zoonotic crisis such as the COVID-19 pandemic, this protective concern may intensify, contributing to elevated stress among owners who fear transmitting disease to their pets.

Conversely, emotional security and companionship pets provide may offset zoonotic fears, potentially explaining the null association between pet-to-owner transmission concern and perceived stress. [63] Prior studies also suggest that established pet ownership is associated with resilience during stressful events, such as pandemics, disasters, and economic recessions, which may further explain why zoonotic concerns did not lead to elevated stress. [24,60] Taken together, these findings highlight the multifaceted nature of the human-animal bond, suggesting that the psychological impact of pet ownership is shaped not only by human-animal interaction and emotional closeness but also by owner’s perceived responsibility, concern for pet vulnerability, caregiving demands, and contextual stressors such as the COVID-19 pandemic.

Several limitations warrant consideration. First, although our analysis was DAG-guided, the cross-sectional design limits causal interpretation. Because all data were collected at the same time point, temporality is uncertain and reverse causation is possible. We emphasize that the causal assumptions were used to guide confounder identification and statistical adjustment rather than to make causal inferences. In addition, we relied on a single, *a priori* causal structure. Different plausible causal structures could yield different adjustment sets and, consequently, different estimates. Relatedly, the assumptions represented in the DAG are not intended to be exhaustive, and residual confounding cannot be ruled out. Nonetheless, presenting these assumptions explicitly provides a transparent rationale for our analytical approach. Second, the examined mediators collectively accounted for only about one-quarter of the overall association, indicating a need for further research to explore additional mediator-related paths, such as pet personality traits and social activities. Third, owner-dog and owner-cat relationships are inherently different, complicating direct comparisons. While DORS and CORS capture species-specific aspects of the human-animal bond using shared core items, differences in item content must be considered when interpreting cross-species differences. Moreover, despite our efforts to standardize these measures using z-scores, standardization does not ensure construct equivalence. Fourth, the 10-item Perceived Stress Scale does not capture the multidimensional nature of perceived stress, and we did not include physiological indicators of stress (e.g., salivary cortisol, heart rate variability, inflammatory markers), limiting our ability to triangulate psychological and physiological stress responses. Finally, all data were self-report and thus subject to measurement errors and reporting bias.

## Conclusion

By integrating a DAG-guided mediation analysis with a comparative evaluation of dog and cat ownership, this study provides a foundation for future research and evidence-based interventions aimed at optimizing the mental-health benefits of companion animals. Our findings indicate that dog and cat ownership differ only marginally in their overall association with perceived stress; however, mediation analyses suggest species-specific patterns, with human-animal interaction and emotional closeness emerging as the most influential mediators for dog and cat ownership, respectively. Overall, the findings are consistent with social support and attachment theories, suggesting that the psychological impact of pet ownership may reflect both interpersonal and affective pathways.

Because a substantial portion of the association between pet ownership type and perceived stress was not explained by the mediators examined, future studies should investigate additional mediator-related paths. These species-specific patterns can help generate hypotheses for longitudinal studies and may guide the development of species-tailored interventions. For example, future work could test whether interventions that promote dog-related social activities, address attachment-related anxiety, and improve access to behavior and financial support are associated with lower stress.
